# The N17 domain of huntingtin as a multifaceted player in Huntington’s disease

**DOI:** 10.3389/fmolb.2024.1527313

**Published:** 2025-01-07

**Authors:** Hyunju Cho

**Affiliations:** Center for Biomolecular and Cellular Structure, Institute for Basic Science, Daejeon, Republic of Korea

**Keywords:** Huntington’s disease, Huntingtin, N17 domain, aggregation, post-translational modification (PTM)

## Abstract

Huntington’s disease (HD) is primarily caused by the aberrant aggregation of the N-terminal exon 1 fragment of mutant huntingtin protein (mHttex1) with expanded polyglutamine (polyQ) repeats in neurons. The first 17 amino acids of the N-terminus of Httex1 (N17 domain) immediately preceding the polyQ repeat domain are evolutionarily conserved across vertebrates and play multifaceted roles in the pathogenesis of HD. Due to its amphipathic helical properties, the N17 domain, both alone and when membrane-associated, promotes mHttEx1 aggregation. Diverse post-translational modifications (PTMs) in the N17 domain alter the aggregation state, thus modulating the cellular toxicity of mHttex1. Furthermore, the N17 domain serves as a nuclear export signal (NES) and mediates the cytoplasmic localization of mHttex1. This review summarizes the four main roles of the N17 domain in regulating HD pathology and discusses potential therapeutic approaches targeting this N17 domain to mitigate HD progression.

## Introduction

Huntington’s disease (HD) is the most common dominantly inherited neurological disorder, and is characterized by progressive involuntary chorea, cognitive dysfunction, psychiatric disturbances, and premature death (Bates et al., 2015). HD is caused by abnormal expansion of CAG (polyQ) repeats in exon 1 of the huntingtin gene. PolyQ repeats longer than 36 are pathogenic and positively correlate with an increased propensity to form intracellular aggregates and increased disease severity (Scherzinger et al., 1997; Gusella and MacDonald, 2000). The intracellular accumulation of pathological huntingtin aggregates impairs the overall proteostasis network and disrupts the structure and dynamics of the endoplasmic reticulum (ER) and mitochondrial membranes (Gidalevitz et al., 2006; Kim et al., 2016; Bäuerlein et al., 2017; Riguet et al., 2021). This eventually leads to the dysregulation of various cellular processes, including transcription, mitochondrial respiration, ER homeostasis, vesicular trafficking, and axonal transport (Kim et al., 2016; Riguet et al., 2021).

The full-length huntingtin protein (Htt) with 23Q contains a total of 3,144 amino acids (348 kDa). Huntingtin plays diverse functional roles in nervous system development, the transport of vesicles containing brain-derived neurotrophic factor (BDNF), and selective autophagy (Saudou and Humbert, 2016). N-terminal fragments containing exon 1 of huntingtin (Httex1) with expanded polyQ repeats, generated by either aberrant splicing or proteolytic cleavage, have been observed in human postmortem brains and mouse HD models (DiFiglia et al., 1997; Lunkes et al., 2002; Sathasivam et al., 2013; Neueder et al., 2017). Moreover, mutant huntingtin exon 1 carrying a polyQ expansion (mHttex1) is sufficient to recapitulate HD-associated phenotypes in animal models (Yu et al., 2003; Southwell et al., 2016; Yang et al., 2020), and is thus widely used as a relevant model for HD biology and pathology.

**FIGURE 1:**
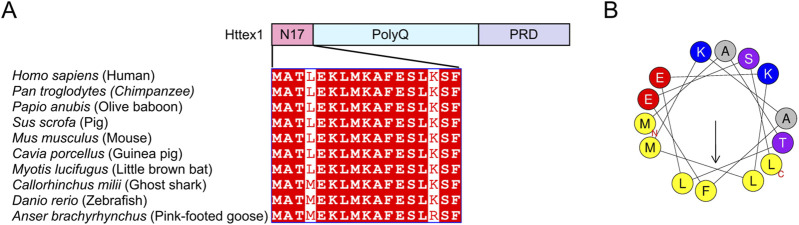
Sequences and amphipathic helical property of the N17 domain of Httex1 **(A)** Sequence alignment of the N17 domain of Httex1. Httex1 is composed of three main domains: N17 domain, polyQ repeat domain, and proline-rich domain (PRD). The sequences of the N17 domain are highly conserved among tested species (Atwal et al., 2007). **(B)** The helical wheel illustrates the distribution of hydrophobic and hydrophilic amino acids in the amphipathic helix of the N17 domain.

**FIGURE 2:**
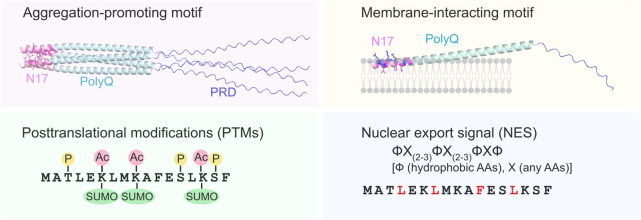
Multi-functional roles of the N17 domain in modulating aggregation and cellular localization of mHttex1. As an aggregation-promoting motif, the hydrophobic residues (highlighted in magenta) in the N17 domain (light pink) form a core hydrophobic interface that may further stabilize polyQ aggregation. The amphipathic N17 domain also interacts with the lipid bilayers, thereby enhancing the local concentration and aggregation of mHttex1. Hydrophobic (magenta) and charged (blue) residues in the N17 domain are located on the interior and exterior surfaces of the membrane, respectively. Three types of PTMs (phosphorylation, acetylation, and SUMOylation) in the N17 domain are known to alter the aggregation state of mHttex1. Furthermore, the N17 domain contains NES-like consensus sequences, ФX_(2–3)_ ФX_(2–3)_ФXФ, where Ф and X are hydrophobic amino acids (highlighted in red) and random amino acids, respectively. In the absence of the N17 domain, toxic mHttex1 aggregates accumulate in the nucleus.

Httex1 comprises three main domains: the N-terminal N17 domain, the PolyQ repeat domain, and the C-terminal proline-rich domain (PRD) (Figure 1A). The 17 amino acid residues in the N17 domain are highly conserved across diverse vertebrate species (Atwal et al., 2007) (Figure 1A). Consistent with this, the N17 domain plays several essential roles in HD pathogenesis by stimulating mHttex1 aggregation and altering its post-translational modifications (PTMs) and cellular localization (Figure 2). In this review, I will focus on these functional roles of the N17 domain and further discuss the implications of N17-targeted therapeutic development for HD.

### N17 domain as an aggregation-promoting motif

The N17 domain is known to enhance the aggregation kinetics of various huntingtin model proteins (Tam et al., 2009; Thakur et al., 2009; Sivanandam et al., 2011; Crick et al., 2013; Vieweg et al., 2021). The N17 domain has an amphipathic helical property (Atwal et al., 2007; Sivanandam et al., 2011) (Figure 1B), and the hydrophobic surface of the amphipathic N17 domain is crucial for the rapid aggregation of mHttex1 (Tam et al., 2009). Consistent with this, the AlphaFold (Jumper et al., 2021) prediction of the tetrameric structure of Httex1-51Q suggests that the hydrophobic residues of the N17 domain interact with each other to form a core hydrophobic interface on the tetramer, thereby potentially stabilizing the self-assembly of mHttex1 (Figure 2). The isolated N17 peptide exhibits the ability to interact with both the N17 and polyQ repeat domains in mHttex1 (Tam et al., 2009). In this proposed mechanism, the N17-N17 and N17-polyQ intermolecular interactions lower the kinetic barrier between the oligomeric and fibril states, thus promoting amyloid fibril aggregation (Shen et al., 2016).

An alternative mechanism is that the hydrogen bonds between the residues in the N17 domain and the polyQ repeat domain enable the stabilization of a long α-helix constituting both domains (Urbanek et al., 2020; Elena-Real et al., 2023). The bifurcated hydrogen bonds between the peptide backbone of F17 or S16 at the *i-4* position and side chains of Q21 or Q20 at the *i* position strengthen the structural coupling between the N17 and polyQ repeat domains, thus stably extending the N17 α-helical content to the polyQ repeat domain through the hydrogen bond network (Elena-Real et al., 2023). The propagation of α-helical content from N17 to polyQ repeat domains increases with the increasing length of the polyQ repeat domain (Elena-Real et al., 2023). Since the N17 domain alone is not sufficient to form aggregation (Thakur et al., 2009), this structural coupling between two adjacent domains could be crucial for accelerating Httex1 aggregation.

### N17 domain as a membrane-interacting motif

The membrane interaction of mutant huntingtin (mHtt) with expanded polyQ repeats appears to be disease-relevant. Both endogenous Htt and the exogenously expressed full-length and truncated N-terminal fragments (∼90 kDa) are present in the membrane fraction of neuron-like clonal striatal cells (Kegel et al., 2000). The N-terminal mHtt fragments are also associated with the brain membranes of both human HD patients and R6/2 mice (Kim et al., 2001; Suopanki et al., 2006). Moreover, the inclusions and fibrils of mHttex1 are known to disrupt various organellar membranes, including nuclear, ER, and mitochondrial membranes. Perinuclear inclusions of mHttex1 interact with the nuclear membrane, destroying nuclear membrane integrity (Waelter et al., 2001; Liu et al., 2015; Riguet et al., 2021). Httex1-97Q-GFP fibrils impinge on the ER membrane and alter the membrane curvature and dynamics (Bäuerlein et al., 2017). N-terminal mHtt is also associated with mitochondrial membranes, potentially contributing to mitochondrial dysfunction in HD (Panov et al., 2002; Orr et al., 2008). Collectively all these results suggest that large fibrillar inclusions impair the membrane integrity of various organelles, thereby contributing to HD pathology.

The N17 domain forms an amphipathic helix in both phospholipid bilayers and DPC micelles (Michalek et al., 2013a; Michalek et al., 2013b; Tao et al., 2019). Similar to other amphipathic helices, hydrophobic residues (L4, L7, F11, L14, and F17) are embedded in the lipid bilayer, and charged residues are located on the membrane surface (Michalek et al., 2013a; Michalek et al., 2013b; Tao et al., 2019) (Figure 2). The N17-anchoring in the membrane is thought to increase the local concentrations of mHttex1, thereby inducing polyQ aggregation on the membrane surface (Michalek et al., 2013b; Tao et al., 2019). Consistent with this, large unilamellar vesicles (LUVs) with a diameter of 100 nm (25% POPS and 75% POPC) enhance the aggregation kinetics of mHttex1 (Pandey et al., 2018). The aggregation-enhancing effects of the N17 domain appear to depend on lipid head charges, saturation levels of phospholipid acyl tails, and membrane curvature (Tao et al., 2019; Beasley et al., 2021; Beasley et al., 2022). Small unilamellar vesicles (SUVs) with anionic lipids (POPG and POPS) dramatically increase Httex1-46Q fibrils, whereas the zwitterionic lipid SUVs with POPC and POPE do not alter the Httex1-46Q fibril content at a 1:10 protein-to-lipid ratio (Beasley et al., 2021). Furthermore, LUVs with saturated DMPC lipids significantly increase Httex1-46Q fibril formation, whereas LUVs with unsaturated DOPC lipids reduce Httex1-46Q fibrils (Beasley et al., 2022). Several studies have suggested that Httex1 preferentially interacts with highly curved membrane surfaces (Chaibva et al., 2014; Tao et al., 2019). Despite the growing evidence of membrane composition-specific effects via the N17 domain, since different organellar membranes have various lipid compositions (van Meer et al., 2008), how the *in vitro* findings can be extended to the organellar-specific membranes remains to be addressed.

### Posttranslational modifications on the N17 domain

PTMs, including phosphorylation, acetylation, and SUMOylation, in the N17 domain alter the hydrophobicity, charge, and secondary structure of the amphipathic helix. These altered physicochemical properties and conformation of the N17 helix affect N17-induced aggregation and membrane interactions, ultimately modulating the cellular toxicity of mHttex1. Thus, PTMs in the N17 domain have been suggested to act as molecular switches in HD pathogenesis, providing an important target for the therapeutic development of HD (Bowie et al., 2018).

Among known PTMs, phosphorylation is the most well-characterized modification. The phosphorylation of the T3, S13, and S16 residues of the N17 domain is generally associated with reduced mHtt toxicity. Consistent with this, decreased levels of mHtt T3 phosphorylation were observed in cellular and mouse HD models, as well as in human HD patient samples (Aiken et al., 2009; Cariulo et al., 2017). Phosphorylation at T3 stabilizes the N17 helical conformation and decreases SDS-insoluble aggregation and fibril formation of Httex1 *in vitro* (Cariulo et al., 2017; Chiki et al., 2017). Despite the relatively lower inhibitory effect of T3D compared to phosphorylated T3 (Chiki et al., 2017), the phosphomimetic T3D mutation is still able to abolish the formation of large inclusions of Httex1-97Q in human H4 glioma cells (Branco-Santos et al., 2017). In contrast, the phosphorylation-deficient T3A mutant was not significantly different from the unmodified Httex1-97Q (Branco-Santos et al., 2017). Furthermore, full-length Htt with phosphomimetic S13D/S16D mutations, but not phosphoresistant S13A/S16A mutations, is known to prevent progressive neuronal dysfunction, mHtt aggregation, and late-onset neurodegenerative pathology *in vivo* (Gu et al., 2009). Similar to phosphorylated S13/S16, the phosphomimetic S13D/S16D mutant delayed the aggregation kinetics of mHttex1 *in vitro* (Mishra et al., 2012). Interestingly, the S13D/S16D mutant showed decreased binding affinity to the membrane, thereby preventing membrane-mediated aggregation (Tao et al., 2019).

Consistent with the notion that increased phosphorylation levels at the N17 domain reduce the toxicity of mHttex1, overexpression of several kinases has been shown to decrease the aggregation of mHttex1 or promote the degradation of mHttex1. Overexpression of the inflammatory kinase IKKβ increases phosphorylation levels at S13 of mHttex1 in ST14A cells, thereby inducing mHttex1 clearance via autophagy (Thompson et al., 2009). Similarly, TANK-binding kinase 1 (TBK1)-mediated phosphorylation at S13 reduces mHttex1 aggregation in an autophagy-dependent manner, leading to decreased neurotoxicity in several HD models (Hegde et al., 2020). Overexpression of the nuclear factor kappa B kinase subunit beta (IKBKB) increases endogenous phosphorylated S13 levels of mHttex1 and reduces Httex1 aggregation in HEK293T cells (Cariulo et al., 2023). Taken together, all previous studies suggest that modulating the expression levels of these specific kinases could be a potential therapeutic strategy to reduce toxic mHttex1 proteins.

Both lysine acetylation at the K6, K9, and K15 residues and N-terminal acetylation have been suggested to modulate the aggregation of mHttex1. A previous mass spectrometry study identified the K9 acetylation of Htt-23Q (1–612) in HEK293T cells (Cong et al., 2011). Single lysine acetylation at K6, K9, or K15 does not play a critical role in the regulation of Httex1-43Q aggregation *in vitro* (Chiki et al., 2017), whereas non-specific acetylation of Httex1-51Q at K6, K9, and K15 with sulfo-N-hydroxysuccinimide (NHSA) prevents fibril formation (Chaibva et al., 2016). In this study, since the *in vitro* aggregation reactions were coupled with acetylation reactions involving NHSA, it remains unclear whether multiple lysine acetylations regulate the aggregation of mHttex1 *in vitro* (Chaibva et al., 2016).

SUMOylation at K6, K9, or K15 of the N17 domain alters the aggregation propensities and toxicity of mHttex1 (Steffan et al., 2004; Subramaniam et al., 2009; O’Rourke et al., 2013). Overexpression of small ubiquitin-like modifier 1 (SUMO1) predominantly SUMOylates K6 and K9 residues of mHttex1 (Steffan et al., 2004). N-terminal SUMO-fused Httex1-97Q also reduced SDS-insoluble aggregation in striatal cells (Steffan et al., 2004). Consistent with this, incubation of Httex1-46Q with SUMO1 significantly decreased the formation of SDS-insoluble aggregates and fibrils *in vitro* (Sedighi et al., 2020). In addition, overexpression of both the Ras homolog enriched in the striatum (Rhes) and SUMO1 increased SUMOylation at K9 and K15 residues and reduced SDS-insoluble aggregates of Httex1-82Q in HEK293 cells (Subramaniam et al., 2009). Given that Rhes interacts more effectively with mHttex1 than with Httex1-WT in both HD cellular and mouse models, and that SUMO modification of mHtt suppresses general transcription, accumulation of SUMOylated mHttex1 has been suggested to cause cytotoxicity despite its reduced aggregation (Steffan et al., 2004; Subramaniam et al., 2009). Reduction of SUMO activity and deletion of SUMO1 ameliorates neurodegeneration in HD fly and mouse models (Steffan et al., 2004; Ramírez-Jarquín et al., 2022).

In addition to SUMO1, overexpression of SUMO2 SUMOylates mHttex1 but enhances SDS-insoluble mHttex1 in HeLa cells (O’Rourke et al., 2013). Furthermore, SDS-insoluble SUMO2-modified proteins largely accumulate in human HD brains compared to controls, suggesting that SUMO2 modification of various proteins could occur during HD progression (O’Rourke et al., 2013). Since most of these studies used the overexpression of SUMO1 and SUMO2 in mammalian cells, the molecular mechanism by which purified SUMOylated mHttex1 directly alters aggregation states remains unclear.

### N17 as a nuclear export signal (NES)

The N17 domain serves as a nuclear export signal (NES) (Maiuri et al., 2013; Zheng et al., 2013). The amino acid sequence of the N17 domain displays a NES consensus-like sequence (ФХ_(2–3)_ФX_(2–3)_ФXФ, where Ф are hydrophobic amino acids (L4, L7, F11, L14 for the N17 domain) and X are random amino acids) (Kutay and Güttinger, 2005; Maiuri et al., 2013; Zheng et al., 2013) (Figure 2). Single mutations in these hydrophobic amino acids or deletion of partial and full-length N17 increased the nuclear localization of Httex1-WT and mHttex1 in various cellular HD models (Cornett et al., 2005; Rockabrand et al., 2007; Zheng et al., 2013). Furthermore, L7S and F11G mutants induce nuclear inclusions of Httex1-72Q in primary mouse cortical neurons (Zheng et al., 2013). Deletion of N17 in full-length mHtt leads to a dramatic acceleration of nuclear aggregation of small mHtt N-terminal fragments in BACHD-ΔN17 (97Q) mouse brains (Gu et al., 2015). BACHD-ΔN17 (97Q) mice exhibit early disease onset and more severe motor and behavioral deficits than BACHD-WT (97Q) mice (Gu et al., 2015). Consistent with this, the induced nuclear accumulation of toxic mHttex1 aggregates in Httex1-ΔN17 (97Q) zebrafish leads to an accelerated HD-like phenotype (Veldman et al., 2015). Therefore, the N17 domain plays an important role in mediating the cytoplasmic targeting of mHtt and in mitigating nuclear toxicity.

N17-mediated nuclear export is regulated by both the CRM1/Exportin 1 receptor and the nucleoporin translocated promoter region (TPR), nuclear basket protein (Cornett et al., 2005; Maiuri et al., 2013; Zheng et al., 2013). The N17 domain alone is shown to be associated with CRM1 and TPR (Cornett et al., 2005; Maiuri et al., 2013). Phosphorylation mimetic mutations (S13D/S16D) and helix-disruption mutations (M8P) reduce the cytoplasmic localization of Httex1 and disrupt the interaction between the N17 domain and CRM1, suggesting that the secondary structure and PTM of N17 could also modulate the cellular localization of Httex1 (Maiuri et al., 2013; Zheng et al., 2013). Furthermore, mHtt interferes with its interaction with nucleoporin TPR, thereby diminishing nuclear export and enhancing mHtt nuclear aggregation (Cornett et al., 2005).

### Potential therapeutic approaches and further directions

The highly conserved N17 domain of huntingtin plays a versatile role in modulating the aggregation and cellular localization of mHtt, thereby contributing to the pathology of HD. Given that N17 acts as an aggregation-promoting motif, direct targeting of the N17 domain could be a promising therapeutic approach for inhibiting mHtt aggregation. A few chaperones, such as TRiC chaperonin and the Hsc70 chaperone, are known to interact with the N17 domain of mHttex1, leading to the suppression of mHttex1 aggregation (Tam et al., 2009; Monsellier et al., 2015). Hsp70s bind their substrates promiscuously and use co-chaperones to enhance substrate selectivity (Mayer and Gierasch, 2019), while TriC requires a complex subunit assembly step (Shahmoradian et al., 2013). However, these requirements may complicate the therapeutic application for HD, with unwanted side effects. Thus, engineering artificial chaperones with enhanced activity and high substrate specificity for N17-mediated mHtt aggregation could be a feasible approach for alleviating the cellular toxicity of mHtt. In addition, a previous study discovered a human antibody that specifically binds to the N17 domain and confirmed its capacity to counteract the aggregation of mHttex1 in cells (Lecerf et al., 2001). Therefore, designing antibodies, short peptides, or small-molecule drugs that directly bind to the N17 domain could be an alternative approach to prevent N17-mediated mHtt aggregation.

In addition to the direct targeting of the N17 domain, indirect modulation of PTM states on the N17 domain of mHttex1 could provide therapeutic potential in HD. Since decreased phosphorylation levels of N17 have been observed in human HD patient samples, and several kinases are known to phosphorylate T3, S13, or S16 residues at the N17 domain, high-throughput screening of compound libraries could enable the identification of small-molecule drugs that restore the phosphorylation levels of the N17 domain (Atwal et al., 2011). However, despite the overall substrate specificity of S/T kinases and tyrosine kinases (Johnson et al., 2023; Yaron-Barir et al., 2024), assessing whether potential kinase activators specifically upregulate the phosphorylation levels of the N17 domain of mHtt could be critical in eliminating any side effects associated with HD therapeutics. In contrast to phosphorylation, the mechanistic understanding of how other PTMs directly regulate N17-induced toxicity lags behind, mainly due to the lack of *in vitro* assays with modified mHttex1 proteins. Therefore, further mechanistic studies on PTMs of the N17 domain could provide therapeutic opportunities to mitigate HD progression by altering PTMs of the N17 domain.
